# Exopolysaccharides from a Scandinavian fermented milk viili increase butyric acid and *Muribaculum* members in the mouse gut

**DOI:** 10.1016/j.fochms.2021.100042

**Published:** 2021-09-22

**Authors:** Takuya Yamane, Satoshi Handa, Momoko Imai, Naoki Harada, Tatsuji Sakamoto, Tetsuo Ishida, Takenori Nakagaki, Yoshihisa Nakano

**Affiliations:** a Center for Research and Development Bioresources, Organization for Research Promotion, Osaka Prefecture University, Sakai 599-8570, Japan; b Department of Applied Life Sciences, Graduate School of Life and Environmental Sciences, Osaka Prefecture University, Sakai 599-8531, Japan; c Institute of Food Sciences, Nakagaki Consulting Engineer and Co Ltd Nishi-ku, Sakai 593-8328, Japan; d Faculty of Human Development, Department of Food and Nutrition Management Studies, Soai University, Osaka 559-0033, Japan; e Department of Applied Life Sciences, Graduate School of Comprehensive Rehabilitation, Osaka Prefecture University, Habikino, Osaka 583-8555, Japan; f Department of Chemistry, Biology and Marine Science, Faculty of Science, University of the Ryukyus, Nishihara, Okinawa 903-0213, Japan

**Keywords:** Viili, Exopolysaccharide, *Muribaculum*, Butylic acid, Intestinal microflora

## Abstract

Starter culture of viili contains lactic acid bacteria belonging to *Lactococcus lactis*. These bacteria secrete large polysaccharides (EPSs) into milk, resulting in a ropy texture of viili. In mouse experiments, a large dose of EPS (5–140 mg/day) has been shown to alleviate severity of artificially induced illness through modulation of the gut microbiota. The present study investigated whether supplementary amounts of EPS affects the gut microbiota of normal mouse. EPS with high glucosamine content (VEPS) was isolated from home-made viili. C57BL/6J male mice fed ordinary diet took 49 ± 1 μg VEPS/day for 28 days by drinking ad libitum tap water containing 8 μg/mL VEPS. The relative abundance of *Muribaculum* increased significantly by VEPS supplementation. The relative abundance of fecal butyric acid decreased in control mice, and VEPS prevented this decrease. These findings indicated that the gut microbiota can be modulated by a small dose of VEPS.

## Highlights

• Bioactivity of viili exopolysaccharide (VEPS) were determined in young male mice.
• Mice drank daily tap water supplemented with VEPS, stressless administration.
• A low dose VEPS modified mildly but significantly the gut microbiota.

## 1 Introduction

Viili is a traditional fermented milk product that originated in Scandinavia (Luo and Deng, 2016, Bakry and Campelo, 2018). To make viili, milk is inoculated with a starter and incubated at about 20 °C for 16–24 h. The viili starter contains *Lactococcus lactis* ssp. *cremoris* and/or *Lactococcus lactis* ssp. *lactis* as slime-forming mesophilic lactic acid bacteria (Kahala et al., 2008). This slime gives viili a ropy texture, and polysaccharides excreted extracellularly by the bacteria, called as exopolysaccharides (EPSs), make the slime. In *Lacococcus lactis*, biosynthesis of these EPSs in viili (VEPS) is carried out by enzymes coded on an *eps* gene cluster in a plasmid (Neve et al., 1988, Zhou et al., 2019).

Various structures of EPS produced by lactic acid bacteria have been determined (Laws et al., 2001, Zannini et al., 2016). Most of these structures are determined for EPS produced by *Lactobacillus and Streptococcus*. Some EPSs contain only one type of monosaccharide (homopolysaccharide) and others consist of more than 2 types of monosaccharide (heteropolysaccharide). EPS is a large polymer (molecular mass of 10^4^–10^7^ Da) consisting of several hundred oligosaccharide units, and the repeat unit is composed of 3–7 monosaccharides linked by α- or β-glycoside bonds. The repeat unit has a principal chain (back bone) and one or more than one branch, which is chemically linked to the back bone. Because determination of the chemical structure of heteropolysaccharide is not an easy task, only a few structures of VEPS are known.

Recently, bioactivities of EPSs produced by lactic acid bacteria have been intensively investigated to find health benefits of EPSs (Korcz et al., 2018, Saadat et al., 2019). For example, in vitro experiments using cells, EPS (50–200 μg/mL in cell culture medium) showed various beneficial bioactivities such as immunoregulation (Kitazawa, Yamaguchi, & Itoh, 1992), anti-tumor activity (Ishiguro et al., 2017), antioxidant activity (Ling et al., 2012), and α-amylase inhibitory activity (Ayyash et al., 2020). In vivo experiments, EPS showed immunoregulation (Vinderola, Perdigón, Duarte, Farnworth, & Matar, 2006) and regulation of intestinal barrier function (Zhou et al., 2018). In these mouse experiments, a large dose of EPS (about 100 mg EPS/kg body weight/day) was administered to muse by oral gavage. Fermented milk products such as yogurts, kefir, and viili are believed to be safe and beneficial to human health. Their beneficial effects are partly due to live lactic acid bacteria (probiotics) and partly due to metabolites produced by the bacteria (Kareb & Aїder, 2019). EPS is one of the metabolites and the heteropolysaccharide content is 50–200 mg/L (Badel, Bernardi, & Michaud, 2011). The intake of EPS in the reported mouse experiments corresponds to 6 g/day in the case of adult human. To find beneficial effects of EPS on human health from the standpoint of functional foods and investigate their mechanism, in vivo experiments using practical dose of EPS are inevitable.

The composition the gut microbiota and host diet is linked closely. Morrison et al. demonstrated that in both female and male mice the reduction of soluble fiber in diet changes substantially the gut microbiome composition, resulting in a loss of taxa within the phylum Bacteroidetes and expansion of Clostrdia and Proteobacteria (Morrison, Jašarević, Howard, & Bale, 2020). Dietary fibers are fermented by the microbiota in the cecum and colon to produce acetate, propionate and butyrate (Koh et al., 2016, Morrison and Preston, 2016, McNabney and Henagan, 2017). These short-chain fatty acids (SCFAs) are absorbed by colonocytes and used as energy source for these cells, and SCFAs transported into circulation act as signaling molecules (Dalile, Van Oudenhove, Vervliet, & Verbeke, 2019). Recently, effects of EPS on the gut microbiota in mouse obesity model (Lim et al., 2017) and liver injury model (Xu et al., 2019) have been reported. In the former model, the relative abundance of *Bacteroides* family and *Akkermansia* family was significantly increased in EPS group mice. In the latter model, pre-administration of EPS for 14 days prevented the gut flora dysbiosis after intraperitoneal injection of lipopolysaccharide and D-galactosamine. It should be noted that in these two experiments 5–140 mg of EPS (corresponding to 12–336 g/day in the case of adult human) was given to each mouse by oral gavage.

The purpose of the present study is to examine whether drinking tap water supplemented with EPS ad libitum affects the gut microbiota and SCFA production in normal mouse fed ordinary diet. First, we isolated EPS from viili (VEPS). The monosaccharide composition and the scanning electron microscopy (SEM) image of VEPS indicated that the present VEPS has a structure different from those reported for *Lactococcus lactis*. Second, the maximum dose of VEPS was determined by comparing the water intake of VEPS group mice with that of control group mice given tap water with no supplement. Finally, tap water containing 8 μg/mL VEPS was administered to C57BL6 mice for 28 days. Mean daily intake of VEPS was 49 ± 1 μg/day. The low dose VEPS increased significantly the relative abundance of *Muribaculum* in the feces. After 28 days, the relative abundance of butyrate in the feces of VEPS group was higher than that of control group.

## 2 Materials and methods

### 2.1 Materials

A viili starter was obtained from Nakagaki Consulting Engineer Co Ltd (Sakai, Japan). The starter contains *Lactococcus. lactis* ssp. *lactis*, *Lactococcus. lactis* ssp. *cremoris*, *Lactococcus. lactis* ssp. *lactis biovar diacetylactis*, and *Leuconostoc meseuteroides* ssp. *Cremoris*. Cow's milk was purchased from a local supermarket.

2-Nitrophenylhydrazine hydrochloride (2-NPH·HCl), *N*-(3-dimethylaminopropyl)-*N'*-ethylcarbodiimide hydrochloride (EDC·HCl), short chain fatty acids, and monosaccharides were purchased from Tokyo Chemical Industry (Tokyo, Japan). All other chemicals (analytical grade) were purchased from Wako Pure Chemicals (Osaka, Japan).

### 2.2 Isolation of EPS from viili

Viili was prepared as follows. The viili starter (1 g) was added to 1 L of cows' milk. After mixing by tumbling, the mixture was left to stand at 22.5 °C for 24 h until the milk solidified. To precipitate proteins, trichloroacetic acid (TCA) was added to the viili (final TCA concentration of 6.7%). The mixture was vigorously stirred at 4 °C for 1 h using a magnetic stirrer, and then the mixture was centrifuged at 12,000×*g* for 20 min at 4 °C. The supernatant was collected and mixed with 2 volumes of ethanol. The mixture was incubated at 4 °C for 12 h, and then the mixture was centrifuged at 12,000×*g* for 20 min at 4 °C. The precipitates were collected and resuspended in distilled water. The suspension was dialyzed against 1 L of distilled water at 4 °C for 1 day using a cellulose membrane with a molecular weight cut off of 10 kDa. Distilled water was exchanged 2 times during dialysis. The dialyzed sample (VEPS) was lyophilized using a freezing dryer (DRW040DA, ADVANTEC, Tokyo, Japan).

### 2.3 Animal experiments

C57BL/6J male mice at 6 weeks of age were purchased from Japan SLC and individually housed in mouse cages. For acclimatization, the mice were fed a normal diet (CE-2, CLEA Japan, Tokyo, Japan) and tap water for 2 weeks. The diet (100 g) contained 4.3 g dietary fibers. After 2 weeks of acclimatization, the mice were assigned to two groups (6 mice per group). The mice in the treatment group (EPS group) were allowed to drink ad libitum tap water containing 8 μg/mL of VEPS. The mice in the control group were allowed to drink tap water. The mice in both groups were fed the normal diet. On the 1st day and 28th day of treatment, feces were collected from individual mice. The feces were kept at −70 °C until use. 2.9. All animal experiments were carried out in accordance with the National Institutes of Health Guide for the Care and Use of Laboratory Animals, and all of the protocols were approved by the Committee for Animal Research at Osaka Prefecture University (permit number 19–161).

### 2.4 Scanning electron microscopy (SEM) of VEPS

A small aliquot of lyophilized VEPS (1 cup of micro spatula) was treated with 4% paraformaldehyde in 5 mL of 0.1 M phosphate buffer (pH 7.4, buffer A) for 8 h at 4 °C. After incubation, the VEPS was precipitated by centrifugation. The precipitates were washed with 5 mL buffer A. The washed VEPS was collected by centrifugation and then treated with 1% tannic acid in 5 mL buffer A at 25 °C for 30 min. The VEPS was precipitated by centrifugation and the precipitates were washed with 5 mL buffer A and then washed with 5 mL distilled water at 25 °C for 30 min. The washed VEPS was dehydrated in an ethanol series: 50%, 70%, 90%, 95%, and finally *tert*-butanol (the alcohol solutions being exchanged every 30 min). The dehydrated VEPS was lyophilized, fixed in stubs on a double-faced metallic tape, and covered with a thin layer of gold using an ion sputter (E-1010, Hitachi, Tokyo, Japan) at 20 mA. The fixed samples were observed using a scanning electron microscope (SU1510, Hitachi, Tokyo, Japan) at an accelerating voltage of 15.0 kV.

### 2.5 Analysis of monosaccharide composition

VEPS (2 mg) was hydrolyzed in 0.1 mL of 2 mol/L sulfuric acid at 120 °C for 2 h. The solvent was evaporated and the hydrolysates were dissolved in 0.26 mL of 0.1 mol/L NaOH (solvent A), and then an aliquot of the solution (80 μL) was applied to a Dionex CarboPac PA1 IC column (4 × 250 mm) (Thermo Fisher Scientific, Waltham, MA). The column was developed at a flow rate of 1 mL/min using the following gradient: 0–5 min, 0% B; 5–35 min, 0–90% B (linear increase). Solvent B was solvent A containing 0.5 mol/L sodium acetate. The effluent was monitored by pulsed amperometric detection using a PAD detector (Dionex ICS-5000, Thermo Fisher Scientific). Authentic monosaccharide solutions (0–100 μM) were used to obtain calibration curves.

### 2.6 SDS-polyacrylamide gel electrophoresis (SDS-PAGE) of VEPS

SDS-PAGE was carried out using precast gels (Any kD precast gel, Bio-Rad) according to the manufacturer's instructions. Proteins were stained with Coomassie Brilliant Blue G-250 using CBB-G250 solution (Bio-Rad, CA, USA). To visualize polysaccharides, periodic acid-Schiff (PAS) staining (Zacharius, Zell, Morrison, & Woodlock, 1969) was performed. Briefly, immediately after the electrophoresis, the gels were fixed in 12.5% TCA for 30 min, rinsed lightly with distilled water, and then immersed in 1% periodic acid (in 3% acetic acid) for 50 min. After periodic acid treatment, the gels were washed in distilled water overnight (water being exchanged 3 times). The washed gels were immersed in fuchsin-sulfite solution (Sigma-Aldrich, MO, USA) in the dark for 50 min and then washed with 0.5% metabisulfite for 10 min. This washing was carried out 3 times. The gels were then washed in distilled water overnight and stored in 5% acetic acid.

### 2.7 Analysis of the composition of gut microbiota by 16S rRNA sequencing

On the 1st day and 28th day, feces were collected individually from mice in the control and VEPS groups. Extraction of DNA from feces, amplification of the V3–V4 region of 16S rRNA (sense primer, 5′-TCGTCGGCAGCGTCAGATGTGTATAAGAGACAGCCTACGGGNGGCWGCAG-3′; antisense primer, 5′-GTCTCGTGGGCTCGGAGATGTGTATAAGAGACAGGACTACHVGGGTATCTAATCC-3′), indexing by sequencing adapters, and sequencing using MiSeq (Illumina, San Diego, CA) were performed as described previously (Harada et al., 2020). Post-processing sequencing data were analyzed using the QIIME2 pipeline (Bolyen et al., 2019). After denoising using Deblur (Hyde, Gonzalez, & Knight, 2017), the filtered sequence data were classified against Silva (v132). Principal coordinate analysis (PCoA) and hierarchical cluster analysis were performed using R software. Bacterial 16SrRNA sequencing data sets have been deposited in DDBJ Sequence Read Archive under the accession number DRA012528.

### 2.8 Analysis of short chain fatty acids

Feces (about 3.0 g) were homogenized in 10 volumes (v/w) of distilled water using a polytron homogenizer, and the homogenate was centrifuged at 3000×*g* for 10 min. An aliquot of the supernatant (1 mL) was further centrifuged at 13,000×*g* for 10 min. The supernatant (500 μL) was transferred to a new 1.5-mL tube and mixed with 500 μL acetonitrile. The mixture was incubated on ice for 10 min and then centrifuged at 13,000×*g* for 10 min. An aliquot of the supernatant (40 μL) was mixed with 135 μL of 50% aqueous acetonitrile and 25 μL of 2.0 mmol/L heptanoic acid (in 50% aqueous acetonitrile). 2-NPH derivatization of SCFAs in this sample (200 μL) was performed according to the method used by Peters et al. (2004). The sample was mixed with 20 μL of 40 mmol/L 2-NPH∙HCl (in 50% aqueous ethanol) and 40 μL of 125 mmol/L EDC∙HCl (in 50% aqueous ethanol containing 1.5% pyridine). The mixture was incubated at 60 °C for 15 min. The reaction was stopped by cooling the mixture on ice. To remove excess ethanol and acetonitrile, the cooled mixture was subjected to evaporation for 30 min using a centrifugal evaporator. Salts and excess reagents in the resultant sample were removed by solid phase extraction using MonoSpinC18 (GL Science, Tokyo, Japan) as follows. The spin column was washed with 300 μL methanol and then equilibrated with 300 μL 0.1% aqueous TFA (solvent C). The sample was diluted with 300 μL solvent C, and the diluted sample was applied to the spin column. The column was washed with 300 μL solvent C twice. The 2-NPH-derivatized fatty acids were then eluted from the monolith with 50 μL of 0.1% TFA in 90% aqueous acetonitrile (solvent D): solvent C = 50:50 (v/v).

The eluted sample was 5-fold diluted with solvent C and a 20-μL aliquot of the diluted sample was applied to an InertSustain C18 column (2.1 mm × 100 mm, GL Science). The column was developed at the flow rate of 150 μL/min according to the following time program: 0–5 min, 10% B; 5–10 min, 10–20% B; 10–50 min, 20–90% B; 50–59 min, 90% B; 59–60 min, 90–10% B. The absorbance at 400 nm was monitored. Chromatograms were analyzed using the chromatography software Clarity (DataApex, Prague, Czech). Calibration curves of the fatty acids were constructed by plotting the peak area ratio of each fatty acid to heptanoic acid against concentration. HPLC analysis was performed using a Prominence HPLC system (Shimadzu, Kyoto, Japan).

### 2.9 Statistical analysis

Data are expressed as means ± S.E. of at least three replicates for each sample. Statistical analyses were performed using Statcel4 software (OMS, Tokyo, Japan). The difference between two groups was evaluated using analysis of variance (one-way ANOVA) followed by unpaired Student’s *t*-test at p < 0.05. For comparison of multiple samples, the Tukey-Kramer test was used.

## 3 Results and discussion

### 3.1 Isolation and characterization of VEPS

VEPS (33.5 mg) was isolated from the viili produced in house from 1 L of milk. Ruas-Madiedo et al. isolated about 70 mg EPS from commercial fermented milk viili (Ruas-Madiedo, Gueimonde, de los Reyes-Gavilán, & Salminen, 2006).

**Fig. 1:**
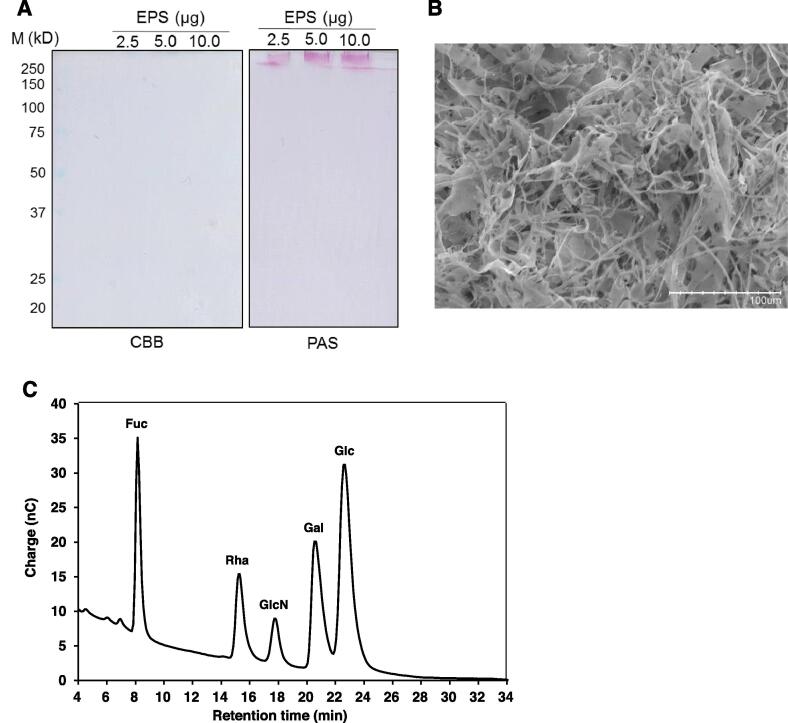
Characterization of VEPS A. SDS-PAGE of VEPS. The left gel was stained with CBB. The right gel was PAS stained. Lane M, pre-stained molecular weight markers. B. SEM image of VEPS. Scale bar, 100 μm. C. Monosaccharide composition of VEPS. Acid hydrolysate of VEPS was subjected to a Dionex CarboPac PA1 IC column and detected by pulsed amperometry. Fuc, fucose (used as an internal standard); Rha, rhamnose; GlcN, glucosamine; Gal, galactose; Glc, glucose.

The SDS-PAGE of the purified VEPS revealed no band stained with Coomassie blue (the left panel of Fig. 1A), indicating that VEPS contained no detectable levels of protein. A relatively broad PAS-stained band (the right panel of Fig. 1A) was detected near the upper edge of the separation gel. When 10 μg of VEPS was applied (the third lane in Fig. 1A), a thin PAS-stained band appeared just below the broad band, indicating that VEPS contained at least two polysaccharides with different molecular sizes.

The SEM image VEPS showed a structure of entangled mixture of long fibers and flakes (Fig. 1B). This structure is distinct from that of EPS from *Lactococcus lactis* F-mou in which a flake-like structure dominates (Nehal et al., 2019), that of EPS from *Lactobacillus plantarum* KX041 in which flakes with rough surface dominates (Xu, Cui, et al., 2019), and that of EPS from *Lactococcus garvieae* C47 in which stick-like and flake-like structures are layered (Ayyash et al., 2020). The fibrous nature of VEPS may be related to the ropy nature of viili.

As shown in Fig. 1C, VEPS contained rhamnose, glucose, galactose, and glucosamine in the molar ratio of rhamnose/glucose/galactose/glucosamine = 3.6/1.0/3.4/5.2. We compared monosaccharide composition of the EPS produced by *Lactococcus lactis* species in Table 1. The EPS produced by *L. Iactis* subsp. *cremoris* SBT 0495 contains rhamnose, glucose, galactose, and phosphorus in the molar ratio of 1.3:1.8:2.1:1.0. (Nakajima, Hirota, Toba, Itoh, & Adachi, 1992). The VEPS isolated in this study contains much higher amount of glucosamine compared to other EPSs. The acetylation level of glucosamine in VEPS is remained to be determined. Elucidation of VEPS structure is important to understand its biological activities.

**Table 1:** Sugar composition of EPS produced by lactic acid bacteria belonging to *Lactococcus lactis*.

Organisms	Culture medium	sugar composition (molar ratio) Glc/Gal/Rha/GlcN	Reference
*Lc. lactis* ssp. *cremoris* SBT 0495	WDM	2.0/2.0/1/0	Nakajima et al., 1992
*Lc. lactis* ssp. *cremoris* LC330	CDM	6.0/3.0/0/2.0	Marshall et al., 1995
*Lc. lactis* strain *cremoris* NIZO B40	WDM	2.3/1.4/1.1/0	Looijesteijn and Hugenholtz, 1999
*Lc. lactis* ssp. *cremoris* B39	WDM	2.0/3.0/2.0/0	van Casteren et al., 2000
Viili starter	cows' milk	1.0/3.4/3.6/5.2	the present study

*Lc*, *Lactococcus*; WDM, whey permeate medium; CDM, cremoris defined medium, Glc, glucose; Gal, galactose; Rha, rhamnose; GlcN, glucosamine.

### 3.2 Effects of the EPS on mice weight and food intake

**Fig. 2:**
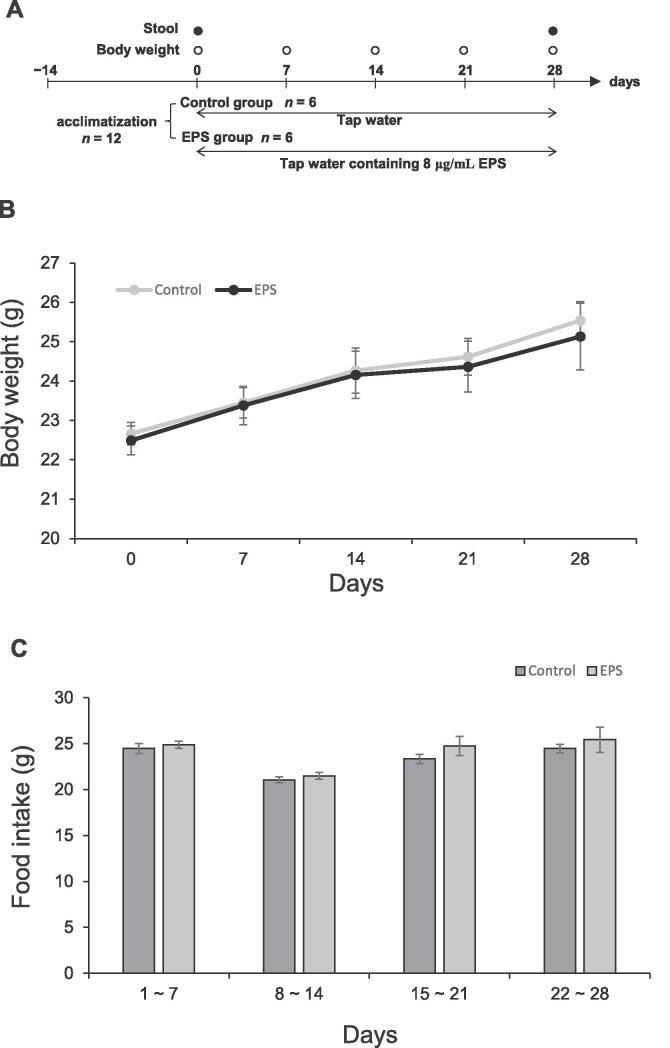
Body weight and food intake A. Schedule of the animal experiments. B. Body weight. Weekly body weight was determined for all mice. C. Food intake. Each mouse was given pre-weighted diet and its remnants after 7 days were weighted to determine weekly food intake. Data are presented as mean ± SE.

The concentration of VEPS in tap water was determined by measuring the water intake of VEPS group mice. The control group mice drank 9.0 ± 0.3 mL tap water/day. When the VEPS concentration was increased up to 8.0 μg/mL, the daily water intake decreased to 6.1 ± 0.2 mL. Based on these results, we determined the maximal acceptable VEPS concentration in tap water to be 8.0 μg/mL. Fig. 2A provides the experimental design. Daily intake of about 50 μg VEPS did not affect the body weight gain (Fig. 2B). Throughout the experiment, the VEPS group mice ate the same amount of food (3.1–3.7 g/day) as the control mice did (Fig. 2C). Because the normal diet contained 4.3 % (w/w) dietary fibers, the mice took 130–160 mg dietary fibers per day. Therefore, the intake of VEPS (50 μg/day) is much lower than that of dietary fibers.

Lim et al. fed C57BL/6J mice with high-fat diet containing 5% (w/w) exopolysaccharides (EPS) isolated from kefir grains and observed the reduction in body weight gain of EPS-treated mice (Lim et al., 2017). In their experiments, daily intake of EPS was 140 mg, about 3000-fold higher dose compared to the present study. The different results on body weight gain indicate the importance of diet used and dose of EPS to investigate effects of EPS on C57BL/6J mice.

### 3.3 Effects of VEPS on mice fecal microbiota

**Fig. 3:**
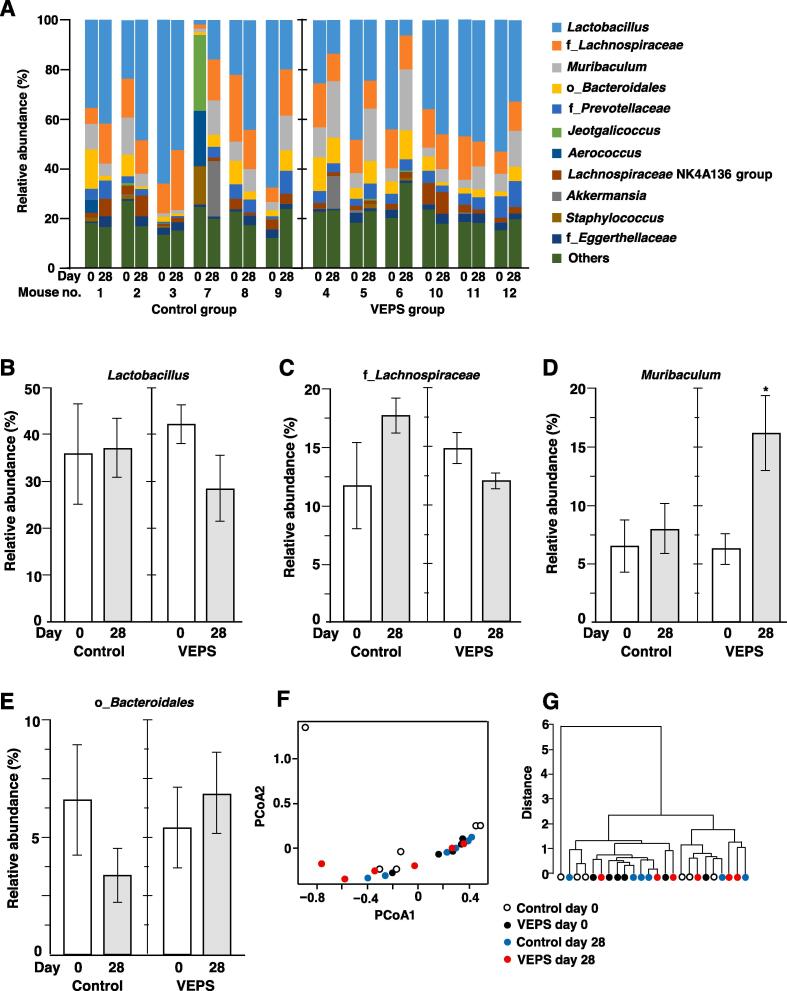
Composition of the fecal microbiota at the genus level A. Relative abundance of operational taxonomic units (OTUs) before (Day 0) and after intake of VEPS (Day 28). VEPS group mice (mouse no. 4–6 and 10–12) drank tap water containing 8.0 μg/mL VEPS for 28 days. Control group (mouse no. 1–3 and 7–9) drank only tap water. B–E. Mean relative abundance of bacteria belonging to *Lactobacillus* (B), *Lachnospiraceae* family (C), *Muribaculum* (D), and *Bacteroidales* order (E), respectively. Data expressed as mean ± SE. Asterisk indicates statistically significant difference (*p* < 0.05). F. Principal coordinate analysis (PCoA) plot. G. The hierarchical cluster analysis. In F and G, each feces sample is shown as a single circle.

Fig. 3A shows the bacterial composition at the genus level of each fecal sample. The dominant genera were *Lactobacillus*, those belonging to *Lachnospiraceae* family, *Muribaculum*, and those belonging to *Bacteroidales* order in all the fecal samples (35–80% of total bacteria) except that collected from mouse no. 7 on day 0, which showed abnormal microbial composition compared to other samples due to unknown reason. Principal coordinates analysis showed no significant clustering of fecal samples based on day and group (Fig. 3F). Phylogenetic tree analysis revealed no clustering of samples (Fig. 3G). These results indicate that small dose of VEPS did not induce a large alteration in the composition of the gut microbiota.

Interestingly, the relative abundance of *Muribaculum* increased in all VEPS-treated mice (Fig. 3A). The mean relative abundance of *Muribaculum* of VEPS group mice increased from 6.3% to 16.2% (Fig. 3D). No significant change was observed for other dominant bacterial groups (Fig. 3B, 3C, and 3E).

*Muribaculaceae* family, previously called as family S24-7, is a dominant bacterial group in the mouse gut (Seedorf et al., 2014, Lagkouvardos et al., 2016, Lagkouvardos et al., 2019). *Muribaculum* is the first described genus belonging to this family. Recently, two species, *M. intestinale* DSM 28989^T^ and *M. gordoncarteri* sp. nov. (DSM 108194^T^), were isolated from faces of conventionally raised C57BL/6J mice (Miyake et al., 2020). These bacterial cells are Gram-stain-negative, strictly anaerobic, lack oxidase and catalase activities, and have genes coding a variety of enzymes involving the degradation of polysaccharides. In colitis mouse model induced by dextran sulfate sodium, the relative abundance of *Muribaculaceae* was negatively correlated with pro-inflammatory cytokines, and positively correlated with the expression levels of tight junction proteins and mucin2 (Yan et al., 2019). Therefore, bacterial species belonging to *Muribaculum* seem to be important for keeping normal conditions of the mouse gut.

The effects of oral gavage of a large dose of EPS on the gut microbiota have been investigated. For example, Xu et al. administered about 8 mg EPS produced by *Lactobacillus buchneri* TCP016 to female BALB/c mice by daily gavage for 14 days (Xu, Aruhan et al., 2019). This EPS treatment did not modulate largely the gut microbiota but significantly alleviated the gut microbiome alterations caused by intraperitoneal injection of lipopolysaccharide/D-galactosamine (acute liver injury model). In an aging mouse model, daily administration of about 1 mg EPS produced by *Lactobacillus plantarum* YW11 by oral gavage prevented the decrease of the gut microbiota diversity of mice injected subcutaneously D-galactose solution (Zhang et al., 2017). These reports suggest that EPS is useful for improvement of the dysbiosis found in these diseases. However, the large dose of EPS is highly problematic in application to food supplement because 1–8 mg EPS/day corresponds to 3–24 g EPS/day in the case of human adult.

### 3.4 Effects of VEPS on mice fecal SCFAs

**Fig. 4:**
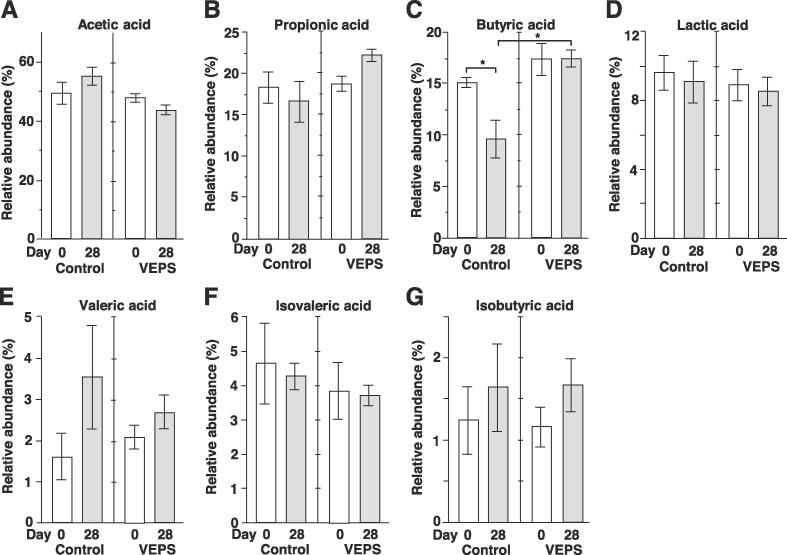
Composition of fecal SCFA and lactic acid. The mean relative abundance of acetic acid (A), propionic acid (B), butyric acid (C), lactic acid (D), valeric acid (E), isovaleric acid (F), and isobutyric acid (G) was calculated for control group mice (Control) and VEPS group mice (VEPS) on days 0 and 28. Data are expressed as mean ± SE. Asterisks indicate statistically significant differences (*p* < 0.05).

We measured SCFAs and lactic acid in feces. Unexpectedly, in control group mice, the relative abundance of butyric acid significantly decreased from 15.0 ± 1.7 % to 9.5 ± 1.3 %, whereas in VEPS group the relative abundance of butyric acid did not changed (Fig. 4C). Other SCFAs and lactic acid showed no significant change after 28 days (Fig. 3A, 3B, 3D–G). Acetic acid, propionic acid, and butyric acid are produced by bacteria metabolizing fiber non-digestible in the gut, and these fecal SCFAs decrease in aged mice (18–20 months) compared to young mice (2–3 months) (Lee et al., 2020). In the present study, mice were 2 months old on day 0, and became 3 months old at the end of the experiments. Therefore, aging is probably not the reason for the decrease in the fecal butyric acid in the control mice. Low dose VEPS seems to prevent the reduction of butyric acid in the mouse gut.

## 4 Conclusion

Exopolysaccharides with new monosaccharide composition (VEPS) were isolated from viili. The highest dose of VEPS (50 μg/day) compatible with drinking ad libitum was administered via tap water to young male mice fed normal diet. VEPS specifically increased the relative abundance of the genus *Muribaculum* in the gut of microbiome. The decrease of fecal butyric acid during feeding was found and it was prevented by VEPS. This is the first report on the effects of low dose VEPS on the gut microbiome of normal young mice.

## Declaration of Competing Interest

The authors declare that they have no known competing financial interests or personal relationships that could have appeared to influence the work reported in this paper.
